# Proteomic Fingerprint of Lung Fibrosis Progression and Response to Therapy in Bleomycin-Induced Mouse Model

**DOI:** 10.3390/ijms24054410

**Published:** 2023-02-23

**Authors:** Lucrezia Principi, Erica Ferrini, Roberta Ciccimarra, Lisa Pagani, Clizia Chinello, Paolo Previtali, Andrew Smith, Gino Villetti, Matteo Zoboli, Francesca Ravanetti, Franco Fabio Stellari, Fulvio Magni, Isabella Piga

**Affiliations:** 1 Clinical Proteomics and Metabolomics Unit, Department of Medicine and Surgery, University of Milano-Bicocca, 20854 Monza, Italy; 2 Department of Veterinary Science, University of Parma, 43122 Parma, Italy; 3 Experimental Pharmacology & Translational Science Department, Chiesi Farmaceutici S.p.A., 43122 Parma, Italy

**Keywords:** lung fibrosis, bleomycin mouse model, proteomics, bottom-up mass spectrometry, nintedanib, Coronin-1A, lactate dehydrogenase B

## Abstract

Idiopathic pulmonary fibrosis (IPF) is a chronic lung disease characterized by the aberrant accumulation of extracellular matrix in the lungs. nintedanib is one of the two FDA-approved drugs for IPF treatment; however, the exact pathophysiological mechanisms of fibrosis progression and response to therapy are still poorly understood. In this work, the molecular fingerprint of fibrosis progression and response to nintedanib treatment have been investigated by mass spectrometry-based bottom-up proteomics in paraffin-embedded lung tissues from bleomycin-induced (BLM) pulmonary fibrosis mice. Our proteomics results unveiled that (i) samples clustered depending on the tissue fibrotic grade (mild, moderate, and severe) and not on the time course after BLM treatment; (ii) the dysregulation of different pathways involved in fibrosis progression such as the complement coagulation cascades, advanced glycation end products (AGEs) and their receptors (RAGEs) signaling, the extracellular matrix-receptor interaction, the regulation of actin cytoskeleton, and ribosomes; (iii) Coronin 1A (Coro1a) as the protein with the highest correlation when evaluating the progression of fibrosis, with an increased expression from mild to severe fibrosis; and (iv) a total of 10 differentially expressed proteins (*p*_adj_-value ≤ 0.05 and Fold change ≤−1.5 or ≥1.5), whose abundance varied in the base of the severity of fibrosis (mild and moderate), were modulated by the antifibrotic treatment with nintedanib, reverting their trend. Notably, nintedanib significantly restored lactate dehydrogenase B (Ldhb) expression but not lactate dehydrogenase A (Ldha). Notwithstanding the need for further investigations to validate the roles of both Coro1a and Ldhb, our findings provide an extensive proteomic characterization with a strong relationship with histomorphometric measurements. These results unveil some biological processes in pulmonary fibrosis and drug-mediated fibrosis therapy.

## 1. Introduction

Idiopathic pulmonary fibrosis (IPF) is a chronic and progressive disease with a median survival time of 2–3 years from diagnosis [1]. The fibrotic lung is characterized by fibroblastic foci as well as the accumulation of collagen in the extracellular matrix (ECM) space. All of these features lead to the degeneration of alveolar architecture and the decline of respiratory function [2]. Up to now, disease diagnosis and the evaluation of its progression have been based solely on clinical (e.g., level of dyspnea, pulmonary hypertension), radiological (High-Resolution Computed Tomography (HR-CT)), and functional variables (e.g., forced vital capacity) [3].

The key events responsible for the initiation of fibrosis occur as a result of an abnormal wound-healing response, driven by alveolar epithelial injury and subsequent secretion of inflammatory mediators (cytokines and chemokines), which leads to platelet activation and inflammatory cell migration. Pro-fibrotic cytokines, such as transforming growth factor β (TGF-β), released by inflammatory cells, play a central role in fibrogenesis, modulating the recruitment and activation of fibroblasts, promoting collagen synthesis and ECM deposition, and driving the differentiation of fibroblasts into myofibroblasts. Nevertheless, the mechanisms underpinning IPF lung tissue remodeling, fibrosis onset, and progression involve complex signaling pathways that are still largely unknown [4]. Currently, there is no cure for IPF, and nintedanib is one of the two antifibrotic drugs approved for its treatment, slowing down the progression of the disease and preserving lung function. The drug is a triple tyrosine kinase inhibitor (TKI) that targets the vascular endothelial growth factor receptor (VEGFR), the platelet-derived growth factor receptor (PDGFR), and the fibroblast growth factor receptor (FGFR) [5]. Further exploration of the molecular mechanisms of action of nintedanib (NINT) is needed and could pave the way to its future use in the treatment of progressive fibrotic lung diseases other than IPF.

During the last decade, high-throughput proteomics technologies have taken huge leaps in order to shed light on the mechanisms of human disease as well as to clarify the action of drugs, and the understanding of IPF lung fibrosis has already started to gain benefits from these approaches, highlighting the overall landscape of ECM proteins [6] and the role of ER and oxidative stress [7,8] in lung tissue fibrosis. Recently, Zheng et al. applied an integrative multi-omics approach combining transcriptomics and proteomics analysis in order to identify novel potential biomarkers of human IPF. They found butyrophilin-like-9 (BTNL9) and plasmolipin (PLLP) genes being downregulated in IPF patients compared to controls and suggested that both genes might play protective roles in IPF, reducing immune response, inhibiting ECM production, and enhancing endothelium regeneration [9]. Konigsberg and colleagues combined four omics datasets (protein-coding RNA, protein, DNA methylation, and noncoding RNA) from IPF and healthy lung tissues to construct a multi-omics network, and their results confirmed previously validated molecules (i.e., MMP7, AGER, COL17A1, TNXB, LAMC3, and RARA-AS1) and pathways already known to be dysregulated in IPF disease [10]. Nevertheless, despite underlining that the use of such a multi-omics strategy can provide a comprehensive characterization of IPF, both studies show a lack of consistency. In fact, Zheng et al.’s observations were based on a particularly small and rather heterogeneous cohort of solely end-stage IPF patients (*n* = 9 IPF patients and *n* = 9 healthy donors) [9], while Konigsberg et al., despite the higher number of patients involved (*n* = 24 IPF subjects and *n* = 14 control subjects) in the study, did not stratify them based on their fibrotic stage/disease severity [10], which is of paramount importance when seeking to better clarify the molecular mechanisms involved in the development and progression of IPF.

Overall, the molecular mechanisms involved in IPF still remain unclear, and their investigation using human samples is not straightforward for two main reasons: on the one hand, the disease can present with a high degree of inter-patient heterogeneity, and on the other hand, surgical lung biopsy is recommended only in a small number of cases with an uncertain diagnosis. As a consequence, there is a reduced number of human tissue samples available for research purposes, and furthermore, there are also difficulties in finding healthy lung counterparts as control samples. Hence, the most frequently used and internationally recognized animal model to investigate IPF and potential therapies is the bleomycin (BLM) mouse model due to its high degree of reproducibility as well as its effectiveness in mimicking many aspects of human IPF [11,12]. While lung tissue from BLM-induced fibrosis mouse models is highly characterized from a histopathological and radiological point of view [13], little is still known about the molecular events involved in the progression of bleomycin-induced pulmonary fibrosis.

Here, a label-free bottom-up proteomics approach based on nano liquid chromatography coupled with electrospray ionization tandem mass spectrometry (nLC-ESI-MS/MS) analysis was used to characterize the proteomic fingerprint of fibrotic lung tissue derived from bleomycin-treated mice. This was conducted in order to elucidate the molecular mechanisms of fibrosis progression and to highlight novel molecular targets modulated by the anti-fibrotic drug nintedanib.

## 2. Results and Discussion

**Figure 1:**
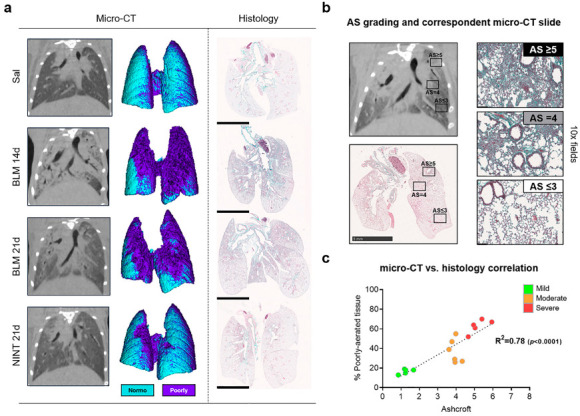
(**a**) For each treatment group, representative coronal 2D micro-CT lung slices are followed by their relative 3D renderings (on the left) and representative whole lung sections are stained with Masson’s trichrome (on the right). In micro-CT renderings, cyan represents the normally aerated tissue while purple regions correspond to poorly aerated tissue; (**b**) Side-by-side comparison between micro-CT and histomorphometric readouts with the identification of different degrees of severity by Ashcroft score. (**c**) A simple linear regression comparing the average Ashcroft score with the % of poorly aerated tissue for each mouse (*p* < 0.0001). Green, orange, and red colors represent the three categories (mild, moderate and severe) defined by the average Ashcroft score value. NINT—nintedanib; BLM—bleomycin; and AS—Ashcroft score.

The present work investigated the molecular hallmarks of fibrosis progression and drug-mediated slowdown while characterizing the proteomic fingerprint of a BLM-induced pulmonary fibrosis mouse model. Prior to lung explantation, all the mice underwent micro-CT lung imaging to non-invasively monitor the progression of pulmonary fibrosis and the anti-fibrotic effect of nintedanib (Figure 1a). Since air represents the natural contrast agent in lung CT imaging, normal lung parenchyma appears darker with respect to more fibrotic portions of the lung. This resulted in it being as dense as the surrounding tissue due to excessive collagen deposition. As expected, a worsening of lung aeration was already observed at 14 days after BLM administration and was highlighted by a decrease in the air content at the end of expiration. A consequent increase in the % poorly aerated tissue, corresponding to the most fibrotic areas, was measured in BLM- and NINT-treated mice compared to healthy mice both at 14 and 21 days (Table S1), especially for mice categorized as severely fibrotic. Overall, however, NINT slowed down fibrosis development by partially restoring lung function when compared to vehicles with a higher percentage of fibrotic tissue (Table S1).

After the last imaging session, lungs were harvested and paraffin-embedded for histological assessment of the fibrosis. Representative whole-lung slides for each group of treatments are reported in Figure 1a. The quantification of fibrosis was based on the Ashcroft score evaluation [14,15]. Representative fields with diverse degrees of fibrotic lesions, along with their match with the corresponding CT scan, have been highlighted in Figure 1b.

As described above, subjects categorized as mild fibrosis present normal lungs with minimal thickening of the alveolar walls at the parenchyma level. Subjects labeled as moderate exhibit a thickening of the alveolar walls without obvious damage of the lung architecture and show heterogeneous areas of individual fibroproliferative masses. The subjects defined as severe show large areas of the confluence of fibroproliferative foci with clear damage and distortion of the pulmonary structure.

The good correlation between micro-CT and the average Ashcroft score, presented in Figure 1c, strongly supports the idea that these two independent methods can be used as a double read-out to describe the overall condition of each sample analyzed and thus to stratify mice for subsequent proteomic analysis.

Lung tissue punches were then collected from histologically healthy parenchyma (control samples) or fibrotic areas (BLM-treated and NINT-treated samples) and analyzed by a label-free, bottom-up nLC-ESI-MS/MS proteomic approach.

There were three different keys to interpret proteomic data: (i) time course after bleomycin treatment (saline, BLM 14 days, and BLM 21 days); (ii) average histological fibrotic grade (mild, moderate, or severe); and (iii) anti-fibrotic treatment effect.

**Figure 2:**
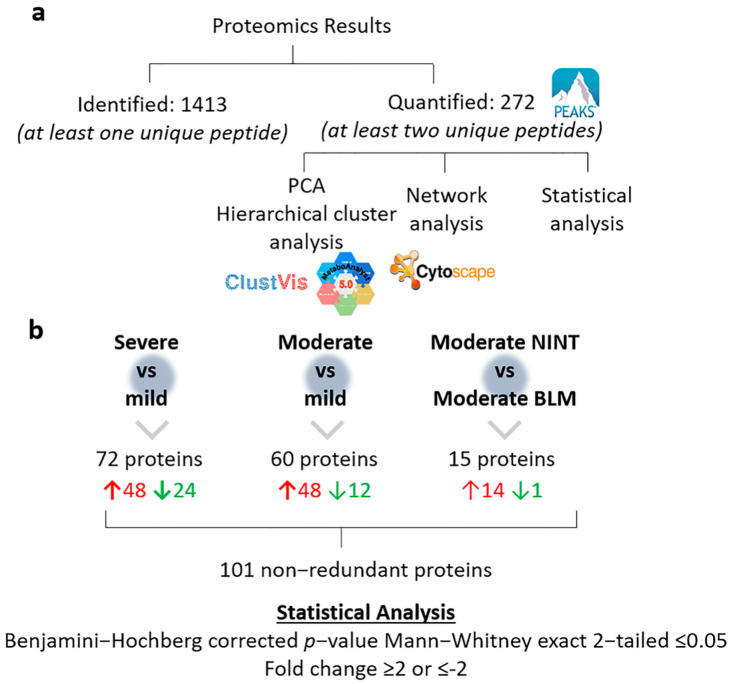
Summary of proteomics results. (**a**) Total numbers of identified and quantified proteins and further data elaboration steps. (**b**) Results of the statistical analysis following pairwise comparisons. PCA—principal component analysis; NINT: nintedanib; and BLM: bleomycin.

A total of 1413 proteins were identified with at least one unique peptide, whereas 272 proteins with at least 2 unique peptides were quantified using a label-free approach. This subset of 272 proteins was further investigated using multivariate data analysis approaches, followed by statistical analysis aimed at performing pairwise comparative proteomic analysis, and finally, functional and pathway analysis (Figure 2a).

### 2.1. Multivariate Data Analysis: Principal Component Analysis and Hierarchical Cluster Analysis

**Figure 3:**
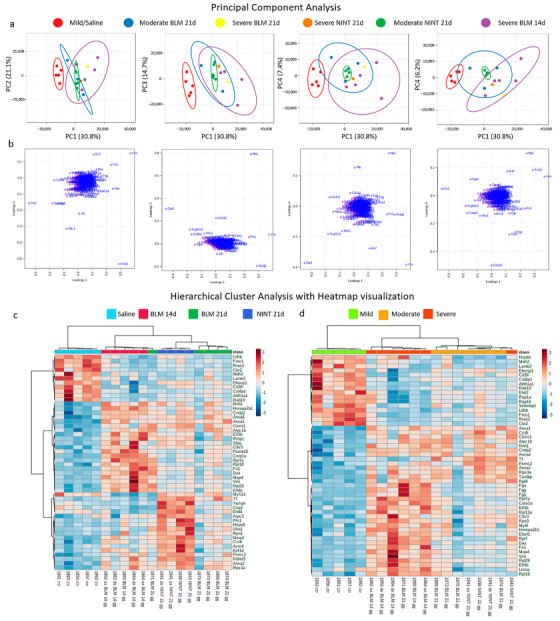
Multivariate Analysis. Principal Component Analysis (PCA): (**a**) 2D score plot between the following PCs, left-to-right: PC1-PC2, PC1-PC3, PC1-PC4, and PC1-PC5. The explained variances are shown in brackets. Pareto scaling is applied to rows, and singular value decomposition (SVD) with imputation is used to calculate principal components. Prediction ellipses are such that, with a probability of 0.95, a new observation from the same group will fall inside the ellipse. *n* = 19 data points; 2D score plots were created with Clustvis. (**b**) Loadings plots; loading plots were created with MetaboAnalyst 5.0. Hierarchical cluster analysis (HCA) with heatmap visualization based on (**c**) sample treatment and (**d**) the average histological grade. Heatmaps were created with MetaboAnalyst 5.0. The data were autoscaled; Pearson was used as a distance measure, and Ward as a clustering method. Heatmaps showed the top 50 features selected by *t*-test/analysis of variance (ANOVA). BLM—bleomycin; NINT—nintedanib; and d—days.

The capability of the label-free, bottom-up proteomic approach to highlight differences in the proteomic fingerprint of non-fibrotic and fibrotic lung tissue samples was initially investigated by performing multivariate analysis, using both unsupervised principal component analysis (PCA, Figure 3a,b) and heatmaps, combined with hierarchical clustering analysis (HCA, Figure 3c,d) of the protein expression data matrix from the 272 quantified proteins. The first 3 components explained 66.6% of the total variance. In particular, the PCA score plots (Figure 3a) of the first three components highlighted that the five biological replicates from saline samples with mild fibrosis (in red) were clustered together and well separated from all the fibrotic samples with moderate or severe fibrosis and treated with bleomycin (14 and 21 days) or with the anti-fibrotic drug nintedanib. However, while investigating further the samples across the fourth (14.7% of the total variance) and fifth (7.4% of the total variance) PCs, the fibrotic samples could also be separated based on their fibrosis severity, going from moderate to severe, while nintedanib biological replicates were clustered together in between moderate and severe samples. Vimentin (Vim), actin (Actg1), hemoglobin (Hba), and fibronectin (Fn1) present in the loading plots (Figure 3b) were positively correlated with fibrotic samples along the first component and negatively correlated with carbonyl reductase [NADPH]2 (Cbr2), uteroglobin (Scgb1a1), collagen alpha-1(IV) chain (Col4a1), and the advanced glycosylation end product-specific receptor (Ager), which was strongly correlated with control samples. Vim and Fn1 overexpression have already been observed in IPF lung tissue [16]. The correlation between these two proteins and fibrotic samples could be explained since an active fibrotic process is ongoing. During the epithelial-mesenchymal transition process (EMT), the specific proteins of the epithelial cells decrease while those typical of the mesenchymal ones, such as actin, increase [16]. Accordingly, the loadings plot showed a positive correlation of Actg1 with fibrotic samples.

In accordance with our results, Lee et al. demonstrated the preventive role of uteroglobin in the development of pulmonary fibrosis in uteroglobin knockout mice (UG-KO) [17]. In particular, they demonstrated that a lack of UG predisposes mice to readily develop pulmonary fibrosis, even when treated with a low dose of bleomycin [17]. Furthermore, similarly to our observation, low levels of Ager and Col4a1 in fibrotic samples have been previously observed in IPF at the proteomic, genetic, and transcriptomic levels [6,18,19,20].

The dataset was further explored by performing HCA using heatmap data visualization. Data autoscaling was applied, and the data were clustered by using the top 50 most statistically significant features (using a *T*-test/Analysis of Variance (ANOVA)) based on treatment (Figure 3c) and on the average histological fibrotic grade (Figure 3d). Interestingly, the HCA results showed that the more appropriate key to interpreting the proteomic mass spectrometry data was the average histological grade of the samples (mild, moderate, and severe) and that sample clusterization was not influenced by the different observation time points of the BLM mouse model (14 or 21 days). The heatmap obtained by clustering the data based on treatment (saline, bleomycin 14 days, bleomycin 21 days, and nintedanib 21 days, Figure 3c) showed that while all five biological replicates of saline samples were under the same tree and clearly separated from all the treated samples (BLM and NINT), on the other hand, samples exposed to BLM treatment for 14 and 21 days were not well-separated. In fact, sample 1871, exposed to BLM for 21 days, was clustered under the same tree as those samples exposed to BLM for 14 days. The answer to this discrepancy was obtained by looking at the average histological fibrotic grade of each sample (Table 1). Sample 1871 was the only one among those exposed to BLM for 21 days to indeed have a severe grade of fibrosis, similar to that of those samples exposed to BLM treatment for 14 days, while the other 4 samples exposed to BLM treatment for 21 days were all characterized by a moderate grade of fibrosis. As a consequence of that, the HCA of the heatmap obtained when stratifying the data based on the average histological grade of fibrosis (Figure 3d), clearly shows that (i) mild samples (light green-colored) were all clustered under the same tree and well separated from all fibrotic samples; (ii) all fibrotic samples were clustered together under a separate tree and further divided into severe (dark orange-colored) and moderate (light orange-colored). In this Ashcroft-based heatmap, sample 1943, with severe fibrosis, is clustered, however, under the tree of moderate fibrosis samples. Indeed, sample 1943 had an average Ashcroft of 4.66 (severe fibrosis, average Ashcroft > 4.5); however, when evaluating the distribution of the single Ashcroft values, no area with a single microscope score of 8 was present, only 6.25% of the area with a single microscope score of 7, and only 9.4% of the area with a single microscope score of 6 (Table 1), for a total of 15.6% of the tissue with scores 6 and 7. However, all the other samples with a severe grade of fibrosis had a total tissue area with a score of 6 or 7, ranging from 24% up to 70%. The score distribution in sample 1943 is hence borderline between moderate and severe samples. It has to be noted that despite tissue punches being collected with a depth of about 2 mm from homogenous areas of fibrotic tissue, the Ashcroft score was evaluated on the superficial histological tissue slice of each FFPE block. Accordingly, the comparisons of the two HCA using heatmap visualization highlighted that the most appropriate parameter to interpret our mass spectrometry proteomic data was the fibrotic severity of tissue.

### 2.2. Pulmonary Fibrosis Progression in Bleomycin-Treated Mice: Mild, Moderate, and Severe

Based on the results of the multivariate data analysis and with the aim of investigating the proteomic alterations involved in fibrosis progression, samples were stratified based on the severity of their fibrosis grade. For this purpose, only saline samples and bleomycin-treated samples were investigated, while all nintedanib-treated samples were excluded from this step.

**Figure 4:**
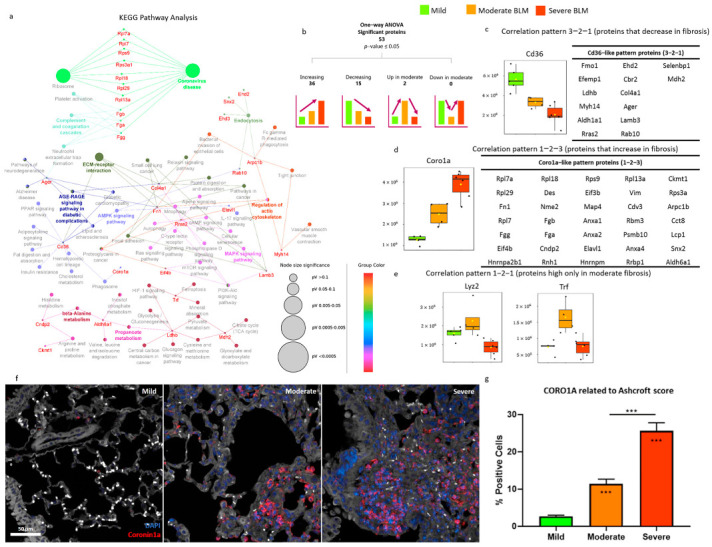
Fibrosis progression in bleomycin-treated mice: mild, moderate, and severe. (**a**) Cytoscape network of KEGG Pathways obtained including in the analysis all the 53 proteins statistically significant in the one-way ANOVA; (**b**) summary of one-way ANOVA statistically significant proteins and with a statistically significant correlation pattern; (**c**) Cd36 boxplot as an example of the correlation pattern 3-2-1 and a list of all the other ANOVA statistically significant proteins with a statistically significant correlation pattern; (**d**) Coro1a boxplot as an example of the correlation pattern 1-2-3 and a list of all the other ANOVA statistically significant proteins with a statistically significant correlation pattern; (**e**) boxplots of the proteins Lyz2 and Trf, characterized by the correlation pattern 1-2-1; and (**f**) representative immunofluorescence microphotographs of lung parenchyma categorized as mild, moderate, and severe (40X magnification; scale bar 50 µm). Nuclei were visualized using DAPI (blue) and Coro1a was stained in TRITC (red); (**g**) histogram shows the percentage of Coro1a-positive cells detected after the immunofluorescence reaction. The data were expressed as the percentage of Coro1a-positive cells and were normalized to the total number of cells segmented and classified as mild, moderate, and severe. Asterisks inside the bars indicate statistical significance versus the corresponding control groups (*** *p* < 0.001), while asterisks above the bars indicate statistical significance between categories (*** *p* < 0.001) (one-way ANOVA). In all boxplots, mild/saline boxes are colored in light green, moderate BLM are colored in light orange, and severe BLM are colored in dark orange. In all boxplots, black dots represent the abundance of the selected features in all samples, yellow diamond represents the mean abundance.KEGG: Kyoto Encyclopedia of Genes and Genomes; ANOVA: Analysis of Variance; Cd36: Platelet glycoprotein 4; Coro1a: Coronin-1A; Lyz2: Lysozyme C2; Trf: Serotransferrin; BLM: bleomycin.

*A one-way* ANOVA, using an adjusted *p*-value cut-off of 0.05, was applied to the dataset, and 53 proteins were found to be statistically significant among the 3 groups (Table S2). In addition to the one-way ANOVA test, correlation analysis was performed using the Pattern Search tool in Metaboanalyst 5.0 against four specific patterns of protein expression: proteins that increase in accordance with the severity of fibrosis (pattern 1-2-3), proteins that, on the contrary, decrease (3-2-1), and proteins that are high (1-2-1) or low (2-1-2) only in moderate fibrosis (Tables S3 and S4). All 53 statistically significant proteins also had statistically significant correlation patterns (*p*-value ≤ 0.05). In particular, 36 proteins had an increasing trend based on the severity of fibrosis, whereas 15 proteins had a decreasing trend, whereas only 2 proteins were higher in moderate fibrosis (Figure 4b–e). Among the 15 proteins with a decreased trend in fibrosis (3-2-1 pattern), hepatic Flavin-containing Monooxygenase 1 (Fmo1) presented the highest correlation pattern. Fmo1 is an enzyme that catalyzes the N-oxygenation of secondary and tertiary amines and is involved in the metabolism of drugs [21]. Hence, the difference observed between mild, moderate, and severe samples is probably related to the bleomycin treatment itself and does not correlate with the mechanisms of fibrosis progression. The second protein with the highest correlation with the pattern 3-2-1 was platelet glycoprotein 4 (Cd36) (Figure 4c). Cd36 is a multifunctional receptor found on the platelet membrane that can bind to distinct types of ligands: thrombospondin, fibronectin, collagen, and other proteins or lipids [22]. The binding of these ligands to Cd36 activates multiple cellular responses, including angiogenesis, an inflammatory response, and fatty acid metabolism [23,24,25]. Numerous studies have investigated the role of Cd36 in lung fibrosis [26,27,28]. Some of these studies have shown that mice with a lack of Cd36 had reduced lung fibrosis, but our results suggest a protective role for Cd36 against lung fibrosis [26,27]. Comparable results were observed in a recent study from 2021, where Wang et al. found a significant decrease in the expression of Cd36 in fibroblasts from fibrotic lungs at both protein and messenger ribonucleic acid (mRNA) levels [28]. Cd36 handles the internalization of collagen in the platelets and its degradation [28]. Hence, the downregulation of Cd36 in moderate and severe fibrosis could be related to the inhibition of collagen catabolism, which has, as a direct consequence, collagen accumulation in the fibrotic lung.

On the other hand, among the 36 proteins with a statistically significant correlation pattern (1-2-3), Coro1a was the one with the highest correlation (Figure 4d). Moreover, it has to be noted that, among all the 53 statistically significant proteins highlighted by the one-way ANOVA, only Coro1a and Rpl29 were statistically significant and with an increased trend in fibrosis in all the comparisons: moderate vs. mild, severe vs. mild, and severe vs. moderate (Table S2). Coro1a, also known as Translational Activator of Cytochrome Oxidase (TACO), is part of the conserved family of coronins, actin cytoskeletal regulators that promote cellular mobility and other actin-dependent processes [29,30]. Recent studies have shown the involvement of Coro1a in inflammatory processes [31,32], in cancer [33,34], and in fibrotic diseases that share common pathways with IPF: chronic classical cardiomyopathy (CCC), characterized by inflammation and myocardial fibrosis; renal interstitial fibrosis (RIF); and cystic fibrosis [35,36,37]. Interestingly, similarly to our observation during lung fibrosis progression, Wu et al. demonstrated that the expression level of Coro1a was significantly higher in the fibrotic tubular cells in chronic kidney disease compared to controls and that the expression level of Coro1a was directly related to fibrosis severity, hence the more severe the fibrosis was, the higher the expression level of Coro1a [36].

Furthermore, the presence of the Coro1a marker on FFPE sections was determined through its expression levels (Figure 4f). A significant increase (*p* < 0.001) in the Coro1a signal was found among the moderate and severe samples compared to the mild group. In agreement with the proteomic data, a statistically significant difference was also detected with the immunofluorescence assay in the severe versus moderate groups (Figure 4g).

Taken together, all these results suggest a possible role for Coro1a as a specific protein indicative of the fibrotic process, irrespective of the affected district (kidney, heart, or lung).

Additionally, Lysozyme C-2 (Lyz) and Transferrin (Trf) were the only two proteins, among the 53 statistically found to be significant in the one-way ANOVA, with a statistically significant correlation pattern and increased expression in moderate fibrosis, while their expression reverted to baseline levels in severe fibrosis (Figure 4e and Table S4). In particular, from the post-hoc analysis, it was observed that Lyz2 was statistically significant and had a higher expression only in the comparison of moderate vs. severe, while Trf had a statistically significant altered expression also in the comparison of moderate vs. mild (Table 1). Interestingly, both proteins were found to be associated with alveolar macrophages.

Lysozymes have primarily a bacteriolytic function, are secreted onto epithelial surfaces, and are found in the primary and secondary granules of neutrophils, as well as the granules of mononuclear phagocytes [38]. Lysozymes are also expressed in both alveolar (which reside in the alveoli) and interstitial (located within the lung parenchymal tissue) macrophages [39].

Moreover, lysozyme overexpression in fibrosis, in particular Lyz1, has already been detected in a bleomycin-induced lung fibrosis rat model [40]. On the other hand, Trf, one of the iron metabolism-related proteins, binds to free iron and transports it into cells. Transferrin-bound iron is imported via the transferrin receptor and exported via the iron exporter ferroportin, both of which are expressed on airway macrophages (AM) and the respiratory epithelium [41]. Pulmonary iron content is therefore tightly regulated, and alterations in iron metabolism have been associated with chronic lung disease, and patients with idiopathic pulmonary fibrosis have been reported to have numerous aspects of dysfunctional iron metabolism. Similarly, pulmonary iron levels increase in bleomycin-induced pulmonary fibrosis in mice [41]. The fibrotic process in the BLM mouse model is characterized by three different phases, which include the inflammatory, proliferative, and maturation phases [13]. Our proteomic results revealed that, in moderate samples, the proteins Lyz2 and Trf were highly expressed, while their expression was lower in severe fibrosis samples. This is most likely due to the fact that lungs with moderate fibrosis present a proteomic profile related to cellular activities involved in tissue remodeling, while in severe fibrotic lesions the main cellular activities are related to ECM deposition and inflammatory cell recruitment.

All 53 proteins found to be statistically significant in the one-way ANOVA were submitted to Cytoscape, and the KEGG pathway database was used as a reference database to gain an overview of the pathways represented in our dataset and, at the same time, map the pathways involved in IPF progression (Figure 4a). Fibrosis progression displayed the alteration of proteins involved in several pathways: ribosome and coronavirus disease; complement and coagulation cascades; advanced glycation end products and receptor for advanced glycation end products (AGE-RAGE) signaling pathway; ECM-receptor interaction; regulation of actin cytoskeleton; endocytosis; tight junction; 5′ adenosine monophosphate-activated protein kinase (AMPK) signaling pathway; RNA transport; pyruvate metabolism; hypoxia-inducible factor-1 (HIF-1) signaling pathway; and phagosome. The ribosome/coronavirus disease pathway was activated, with all the proteins (Rpl7a, Rpl7, Rps9, Rps3a1, Rpl18, Rpl29, and Rpl13a) being all overexpressed (based on post-hoc analysis) in severe fibrotic samples compared to mild. Interestingly, post-hoc analysis highlighted that Rpl7a, Rpl7, Rps9, and Rpl13a were also overexpressed in the comparison of severe to moderate, while Rpl18 and Rps3a1 were overexpressed in the comparison of moderate to mild. The ribosomal protein Rpl29 was the only one with a statistically significant increasing trend in all comparisons from mild to severe fibrosis (moderate-mild, severe-mild, and severe-moderate). Ribosomal proteins are involved in the regulation of apoptosis, cell proliferation, neoplastic transformation, cell migration, and invasion, as well as tumorigenesis [42]. Dysregulated cell proliferation (i.e., fibroblast cells) is one of the key hallmarks of fibrosis, and the overexpression of the ribosome pathway in fibrotic tissues has already been observed in human IPF samples [6]. Similarly, complement and coagulation cascades, platelet activation, and neutrophil extracellular trap formation pathways were also activated and shared the proteins fibrinogen alpha, beta, and gamma chains (Fga, Fgb, and Fgg), which were all overexpressed in severe fibrotic samples (severe-mild and severe-moderate) as previously observed [43,44].

### 2.3. Proteomic Characterization of the Bleomycin-Induced Mouse Model of Pulmonary Fibrosis

The bleomycin-induced lung fibrosis mouse model (C57BL/6) with double OA administration of BLM has already been characterized from a histological and radiological perspective. However, the proteomic characterization of the model is still lacking but necessary in order to better understand the molecular mechanisms involved in the fibrotic process [13]. Among all the 53 statistically significant proteins highlighted in the one-way ANOVA, 22 proteins (Coro1a, Rpl7a, Des, Fn1, Rpl29, Fgg, Fga, Rpl7, Hnrnpa2b, Eif4b, Fgb, Rps9, Map4, Eif3b, Rpl13a, Rrbp1, Elavl1, Vim, Hnrnpm, Psmb10, Aldh6a1, and Rbm3) were overexpressed, while 3 proteins (Trf, Lyz2, and Myh14) were under expressed in the comparison of severe vs. moderate. All these proteins together represent the proteomic signature of fibrosis progression, from moderate to severe fibrosis. On the other hand, 15 proteins (Coro1a, Anxa1, Rpl29, Cndp2, Nme2, Rpl18, Rnh1, Anxa2, Lcp1, Snx2, Anxa4, Cct8, Arpc1b, Ckmt1, and Rps3a1) were overexpressed, while 13 proteins (Ldhb, Fmo1, Rras2, Cd36, Cbr2, Aldh1a1, Efemp1, Rab10, Col4a1, Ehd2, Lamb3, Selenbp1, and Mdh2) were under expressed in both the comparisons moderate vs. mild and severe vs. mild. Hence, all these 28 proteins together represent the proteomic fingerprint of fibrosis onset from a non-fibrotic (mild) to a fibrotic (severe and moderate) state.

Further statistical analysis was conducted focusing on pairwise comparisons (*p*-value_adj_ ≤ 0.05 and with a fold-change ≥ 2 or ≤−2) (Table S5). In particular, 60 proteins (48 up-regulated and 12 down-regulated) were found to be altered in moderate BLM vs. mild, while 72 proteins were altered in the comparison of severe BLM vs. mild (48 up-regulated and 24 down-regulated) (Figure 2b). Among them, 22 proteins were differentially expressed only in moderate BLM/mild, 34 were differentially expressed only in severe BLM/mild, and 38 were shared proteins. In order to perform functional enrichment analysis, the gene names associated with the proteins differentially expressed in the pairwise comparisons were imported into the String-db tool. In particular, functional enrichment analysis of the 38 common proteins between the two comparisons showed a protein-to-protein interaction (PPI) network with 38 nodes and 63 edges and a PPI enrichment *p*-value of 1.06 × 10^−9^. The blood coagulation fibrin clot formation (Fga, Fgg, and Fgb) biological process was enriched (strength 2.4) as well as myofibroblast tissue expression (strength 2.06—Eln and Fn1 genes). Elastin (Eln) and Fn1 are proteins secreted by different subtypes of stromal cells and were overexpressed in both moderate and severe fibrotic samples. On the other hand, from the PPI network obtained from the 22 proteins differentially expressed only in the moderate/mild comparison, emerged the enrichment of the biological processes associated with the regulation of reactive oxygen species biosynthesis (strength 1.59; Ddah2, Rac1, Hsp90ab1, and Eef1a1), heat shock factor 1 (HSF1) activation (strength 2.4; Hsp90ab1 and Eef1a1), the AGE-RAGE signaling pathway (strength 1.47; Col4a2, F3, and Rac1), embryonic fibroblasts (strength 1.19; Hsp90ab1, Hspa8, Uba1, Rpn1, and Eef1a1), and fibroblast tissue expression (strength 1.09; Hsp90ab1, Hspa8, Uba1, Rpn1, Sptbn1, and Eef1a1). Heat shock proteins (HSP) are a category of stress proteins and the HSP90 family is the most abundant of the HSPs, regulating myofibroblast differentiation and promoting ECM and collagen synthesis as well as production [45]. Similarly to our results, the activation of HSP90 was observed both in patients with IPF as well as in BLM-treated mice, in which it could have a regulatory function on stromal cells [46].

The 34 proteins differentially expressed only in the severe/mild comparison showed a PPI network with 34 nodes and 22 edges and a PPI enrichment *p*-value of 9.24 × 10^−5^. This PPI network showed the enrichment of bronchiolar epithelium tissue expression (strength 2.64), with Cyp2f2 and Scgb1a1 proteins being both downregulated in severe fibrosis. These data suggest a modification of the secretive function of bronchiolar epithelium that is mandatory for the protective barrier and preservation of proper airway function [47].

Furthermore, our proteomics results on the BLM mouse model were compared with two recent multi-omics human studies in order to highlight similarities and differences in terms of protein expression between mice and human subjects (Table S5) [9,10]. The 78 proteins found to be commonly differentially expressed in both the transcriptomics and proteomics datasets in the Zheng et al. study were compared to our results [9]. Ager, Selenbp1, and Limch1 were the only three proteins shared between ours and the dataset of Zheng et al. All three proteins were downregulated only in the comparison of severe vs. mild, in accordance with what was observed for IPF end-stage patients [9]. Interestingly, both Selenbp1 and Limch1 were included by Zheng et al. in the thirteen potential marker gene list with the most significant fold changes and adjusted *p* values [9]. These three proteins were also observed in the study of Konigsberg et al. having the same downregulated expression, with the exception of Limch1, which was upregulated in the poly RNA-seq dataset. A total of 56 proteins were in common between our study and that of Konigsberg et al., with Aldh1a1, Ager, Edh2, Cd36, Ehd4, Selendp1, Lamc2, and Ctnnd1 being downregulated while Vim, Hspa8, Des, Rps3, Ldha, Rps18, Khsrp, and Palld, were upregulated, respectively, in both mouse and human proteomics studies. It has to be noted that Ager, Ehd2, Ehd4, Selendp1, and Ctnnd1 were all coherently downregulated in both mouse and human proteomics and transcriptomics datasets [9,10]. These results represent a step forward in the proteomic characterization of the bleomycin-induced pulmonary fibrosis mouse model.

### 2.4. Proteomic Characterization of Nintedanib-Mediated Fibrosis Slow-Down

**Figure 5:**
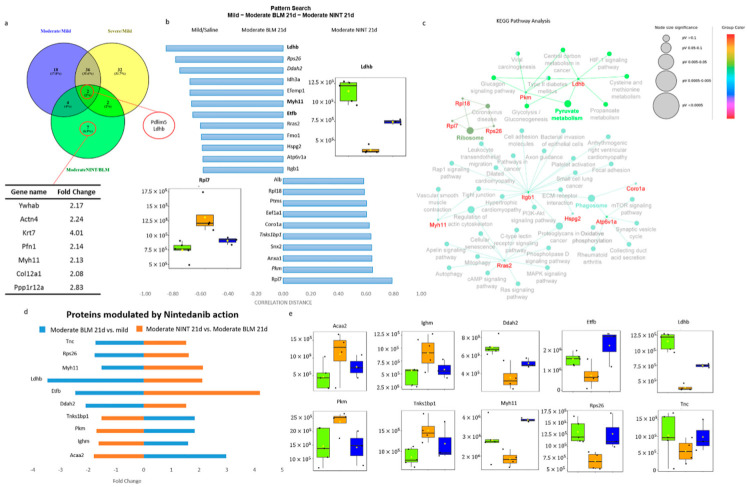
Nintedanib-mediated fibrosis slowdown. (**a**) Venn diagram comparing statistically significant proteins (*p*-value_adj_ ≤ 0.05, Fold Change ≥ 2 or ≤−2) in the comparisons of moderate BLM vs. mild/ severe BLM vs. mild/moderate NINT 21d vs. moderate BLM 21d; the two proteins common to all pairwise comparison are circled in red; and the even proteins are statistically significant (*p*-value_adj_ ≤ 0.05, fold change ≥ 2 or ≤−2) only in the comparison moderate NINT 21d vs. moderate BLM 21d are listed in a table. (**b**) Pattern search analysis of the proteins with a statistically significant (*p*-value ≤ 0.05) correlation pattern and modulated (increased or decreased) by NINT treatment in the comparison between mild (green), moderate BLM 21d (orange), and moderate NINT 21d (blue), the correlation distance value in the bar chart is expressed based on the pattern search 1-2-1. (**c**) Cytoscape network of KEGG pathways obtained including the analysis of all 22 proteins with a statistically significant correlation. (**d**) Ten proteins with a reversing fold change in the comparison between moderate-BLM 21d vs. mild and moderate-NINT 21d vs. moderate-BLM 21d (*p*-value_adj_ ≤ 0.05, FC ≥ 1.5 or ≤−1.5). (**e**) Boxplots of the ten proteins. In all boxplots, mild/saline boxes are colored in green, moderate BLM 21d in orange, and moderate NINT 21d in blue. BLM—bleomycin; NINT—nintedanib; and d—days. In all Boxplots, black dots represent the abundance of the selected features in all samples, yellow diamond represents the mean abundance.

The molecular mechanisms of action of nintedanib and its ability to slow down the progression of BLM-induced lung fibrosis were explored by comparing samples treated with NINT (NINT21d) with samples exposed to BLM for 21d; all samples in both groups had moderate fibrosis. Fifteen proteins were found to be differentially expressed in this pairwise comparison, with 14 up-regulated and 1 down-regulated (Figure 2b). In particular, 7 proteins were altered only in the comparison of Moderate NINT 21d/Moderate BLM 21d, whereas 2 proteins were common to all the three comparisons (Ldhb and Pdlim5) (Figure 5a,b). The pattern search tool of MetaboAnalyst v.5.0 was used in order to detect proteins with a statistically significant correlation pattern (*p*-value ≤ 0.05) with an increased or decreased expression (patterns 1-2-1 or 2-1-2) only in NINT 21d samples compared to mild and moderate BLM 21d. A total of 22 proteins were highlighted (Figure 5c). Among those proteins, only lactate dehydrogenase b (Ldhb), myosin heavy chain 11 (Myh11), and electron transfer flavoprotein subunit beta (Etfb) were statistically significant in the pairwise comparison moderate NINT 21d vs. moderate BLM 21d (*p*-value_adj_ ≤ 0.05 and FC ≥ 2 or FC ≤ −2). Rps26, Ddah2, Tnks1bp1, and Pkm were also statistically significant but with an FC ≥ 1.5 or FC ≤ −1.5 (Figure 5c). These 22 proteins were used to build the KEGG Pathway network (Figure 5d), underlining the alteration of pyruvate metabolism, oxidative phosphorylation, proteoglycans in cancer, the Mitogen-activated protein kinase (MAPK) signaling pathway, and the ribosome. Lowering the FC to 1.5 in both comparisons, moderate BLM 21d vs. mild and moderate NINT 21d vs. moderate BLM 21d, ten proteins (Tnc, Rps26, Myh11, Ldhb, Etfb, Ddah2, Tnks1bp1, Pkm, Ighm, and Acaa2) were inverted in their trend by the action of NINT (Figure 5e,f). Among them, Myh11 was the only one statistically significant within the FC range ≥ 2 or ≤−2 and included in the short list of 7 proteins specific for the comparison of moderate NINT vs. moderate BLM (Figure 5b). Myh11 was down-regulated (FC, −1.51) in the comparison of moderate BLM vs. mild, while it became up-regulated in the comparison of moderate NINT 21d vs. moderate BLM 21d (FC: 2.13). Myh11 is a protein that participates in muscle contraction, tight junction, and regulation of the actin cytoskeleton (Figure 5d) and converts chemical energy into mechanical energy through the hydrolysis of Adenosine Triphosphate (ATP) in Adenosine Diphosphate (ADP) [48]. Previous studies have demonstrated the downregulation of Myh11 in several types of cancers. A recent study demonstrated its downregulation even in lung cancer, highlighting its potential role as a novel drug target and prognostic indicator [49,50,51]. The molecular mechanisms of Myh11 remain, however, still unclear. The downregulation of Myh11 in the moderately fibrotic tissue suggests a decreased utilization of ATP by myosin, possibly leading to an accumulation of ATP in the cells. Both hypoxic conditions and inflammation, which have been hypothesized to be related to fibrosis formation, lead to increased extracellular ATP. Based on our results, Myh11 might be involved in this process [52]. It should be noted that in our results, Myh11 appeared to be statistically significant in the comparison of severe vs. mild but with an FC of only 1.22, while in the comparison of severe vs. moderate had an FC of 1.8. Hence, while Myh11 was downregulated in moderate fibrosis, on the other hand, its expression was restored in severe fibrosis (Figure S1). Nevertheless, the molecular mechanisms involved in Myh11 alteration due to fibrosis progression, as well as the modulatory effect of nintedanib, have yet to be further explored and clarified.

**Figure 6:**
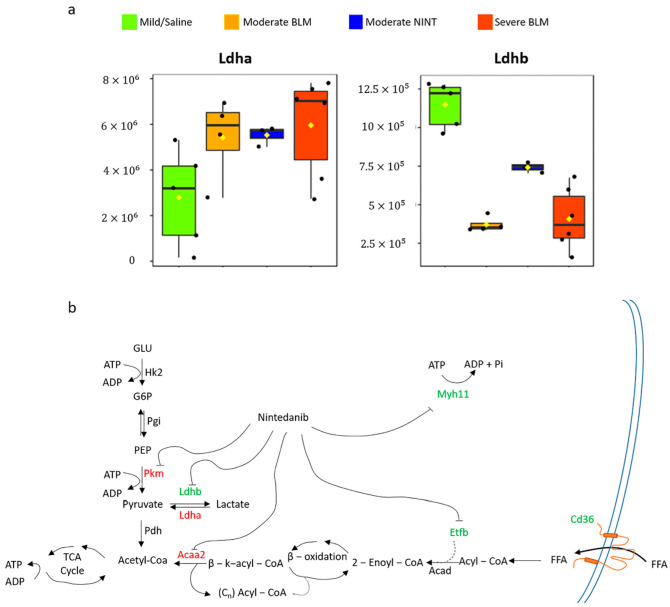
(**a**) Boxplots of lactate dehydrogenase A (Ldha) and lactate dehydrogenase B (Ldhb) in mild (green), moderate BLM (orange), moderate NINT (blue), and severe BLM (dark orange). Black dots represent the abundance of the selected features in all samples, yellow diamond represents the mean abundance. (**b**) Map of the pathways altered in moderate fibrosis and proteins modulated by nintedanib action (*p*-value_adj_ ≤ 0.05, FC ≥ 1.5 or ≤−1.5). The proteins upregulated and those downregulated in moderate fibrosis compared to mild are colored in red and green, respectively. BLM—bleomycin; NINT—nintedanib.

Ldhb was the only protein being both statistically significant with an FC ≥ 2 or FC ≤ −2 shared among all the three pairwise comparisons as well as reverted by nintedanib action (Figure 5a,b). Lactate dehydrogenases, both A and B, are enzymes involved in the conversion pyruvate-lactate: Ldha has a higher affinity for pyruvate and converts pyruvate to lactate, as well as nicotinamide adenine dinucleotide (NAD) + hydrogen (H) (NADH) to NAD^+^, in anaerobic conditions; whereas Ldhb has a higher affinity for lactate, converting lactate to pyruvate, as well as NAD^+^ to NADH, in aerobic conditions. Previous studies have demonstrated the accumulation of lactic acid in IPF lung tissues compared to controls [53]. Our results showed that in bleomycin-induced IPF samples (both moderate and severe), Ldhb was down-regulated while Ldha was up-regulated and, interestingly, despite their similar structure, nintedanib modulated only Ldhb expression (Figure 6a). The dysregulation of lactate metabolism, the cells involved, and the exact mechanisms of action remain yet to be clarified. Danforth et al., in a recent study of 2021, have observed the alteration of lactate metabolism in human Alveolar type II Epithelial Cells (AEC2), and they hypothesize its central involvement in the development and progression of IPF [54]. Although the conversion of pyruvate to lactic acid takes place in physiologically low oxygen tension conditions, it can also still occur in aerobic conditions, known as the Warburg effect, which is typically observed in cancer cells [55]. Recently, a “reverse Warburg effect” has been proposed as a factor contributing to the pathogenesis of fibrosis, suggesting that aerobic glycolysis takes place in the fibroblasts while the secreted glycolytic metabolites influence the behavior of other cell types (such as epithelial cells and macrophages) [56].

Moreover, our results highlighted the deregulation of other proteins (Pyruvate kinase (Pkm), 3-ketoacyl-CoA thiolase (Acaa2), Etfb and Cd36) related to glycolytic pathway, β-oxidation of fatty acids and Tricarboxylic acid (TCA) cycle, suggesting the alteration of ATP metabolism in moderate fibrosis and their modulation by nintedanib (Figure 6b). Interestingly, the expression of Pkm, Acaa2, and Etfb, similar to what was observed for Myh11, was reverted in severe fibrosis (Figure S1).

## 3. Materials and Methods

### 3.1. Reagents and Solutions

Trifluoroacetic acid (TFA), ammonium bicarbonate, trypsin from porcine pancreas (Proteomics Grade, BioReagent, Dimethylated), dithiothreitol (DTT), iodoacetamide (IAA), and formic acid (FA) were purchased from Sigma-Aldrich (Sigma-Aldrich Chemie Gmbh, Buchs, Switzerland). HPLC-grade water, acetonitrile (ACN), ethanol, and toluene were purchased from Honeywell (Honeywell Research Chemicals Riedel-de-Haën™, Seelze, Germany). RapiGest SF surfactant was purchased from Waters Corporation (Waters, Milford, MA, USA). ZipTips were purchased from EMD Millipore (Billerica, MA, USA).

### 3.2. Experimental Animals

The study was conducted using male inbred C57BL/6J mice (Envigo, San Pietro al Natisone, Udine, Italy) aged 7 to 8 weeks. Prior to use, mice were acclimated to the local vivarium conditions (20–24 °C room temperature; 40–70% relative humidity; 12-h light-dark cycle) for at least 5 days, having free access to standard rodent chow and softened tap water. All procedures were in compliance with the principles outlined in the European Directive 2010/ 63 UE, Italian D.Lgs 26/2014, and the revised “Guide for the Care and Use of Laboratory Animals” (National Research Council Committee, US, 2011) [57]. Animal studies were performed in an AAALAC (Association for Assessment and Accreditation for Laboratory Animal Care) certified facility at Chiesi Farmaceutici and authorized by the Italian Ministry of Health with protocol number 809/2020-PR and by the internal AWB (Animal Welfare Body). A visual analog scale (0–10) for pain assessment was assessed daily by a designated veterinarian or trained technicians. VAS ≥ 6 and/or body weight loss ≥ 20% were considered humane endpoints, as were signs of dyspnea or apathy evaluated by a designated veterinarian.

### 3.3. Bleomycin and Nintedanib Administration

**Figure 7:**
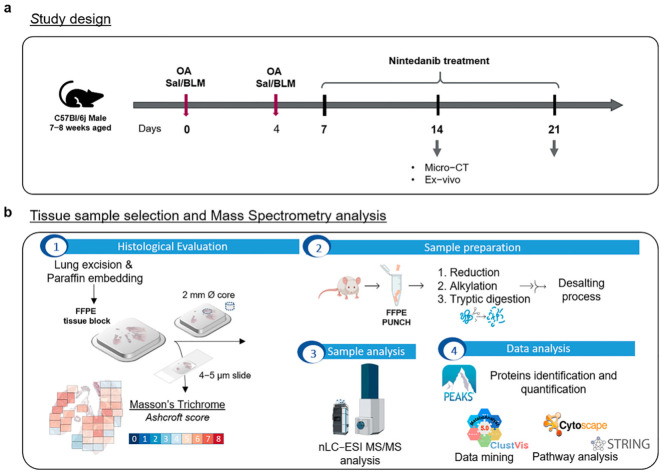
Experimental workflow of all the processes involved in sample collection, preparation, and data analysis. (**a**) Study design for control, BLM, and NINT treatment groups in C57BL6 mice. (**b**) Tissue sample selection and mass spectrometry analysis: (1) lung tissue histological evaluation, (2) sample preparation, (3) sample analysis, and (4) data analysis. BLM—bleomycin; OA—oropharyngeal aspiration; NINT—nintedanib; CT—computed tomography; nLC-ESI-MS/MS—nano-scale liquid chromatographic tandem mass spectrometry; and FFPE—formalin-fixed paraffin-embedded.

Pulmonary fibrosis was induced through oropharyngeal aspiration (OA) [13,58] of 10 μg/mouse bleomycin hydrochloride (Baxter, BLM group) diluted in 50 μL of saline, while vehicle mice received only 50 μL saline (Saline group). The OA procedure was performed at days 0 and 4 in mice lightly anesthetized with 2.5% isoflurane. After induction, high-calorie dietary supplements (recovery gel from Dietgel) and sterile sunflower seeds were added daily to the standard rodent chow in order to reduce body weight loss. At day 7, BLM-treated mice were randomly divided into 2 subgroups, receiving either nintedanib (60 mg/kg/day, Carbosynth Limited, Compton, UK) dissolved in Tween80 0.05% in saline (NINT group) or vehicle (Tween80 0.05% in saline), by gavage, daily for 2 weeks (Figure 7a).

### 3.4. Micro-CT Lung Imaging

Prior to lung excision for proteomic analyses, micro-CT imaging was performed with a Quantum GX Micro-CT (PerkinElmer, Inc., Waltham, MA, USA). The system was calibrated monthly with standard phantoms to check the noise, uniformity, low contrast, and resolution [59]. Images were acquired with an intrinsic retrospective two-phase respiratory gating technique with the following parameters: 90 KV, 88 μA over a total angle of 360° for a total scan time of 4 min. The “high speed” scan mode resulted in two 3D datasets corresponding to the two different phases of the breathing cycle (inspiration and expiration), but the images and data reported in this work refer to the end-expiratory phase. An automated deep learning (DL)-based model previously published by Vincenzi et al. [60] was applied to rescale CT scans into Hounsfield units (HU), setting −1000 HU as the density of air and 0 HU as the density of water, and for the segmentation of the total lung volumes. Pre-clinical HU density ranges [61] were then applied to segmented lungs for the quantitative assessment of parenchymal lesions. Normo-aerated tissue [−860 HU; −435 HU] and poorly-aerated tissue [−435 HU; +121 HU] were defined and expressed as % of total lung volumes. Finally, 3D renderings were generated by using the Analyze software (Analyze 12.0; Copyright 1986–2017, Biomedical Imaging Resource, Mayo Clinic, Rochester, MN, USA).

### 3.5. Histological Assessment of Lung Fibrosis

Following the in vivo procedures, subsets of saline-, BLM-, and NINT-treated mice were culled for the histological assessment of pulmonary fibrosis. The lungs were removed and inflated through the trachea by gentle infusion with 0.6 mL of 10% neutral buffered formalin and fixed for 24 h. Samples were then dehydrated in a graded ethanol series, cleared in xylene, and paraffin-embedded. Sections of 5 μm thickness were cut with a rotary microtome (Slee Cut 6062, Slee Medical, Mainz, Germany) and stained with hematoxylin and eosin (H&E) and Masson’s trichrome (Figure 7b). An immunofluorescence assay was performed to detect, in situ, the Coronin-1A (Coro1a) protein. For this purpose, an anti-Coro1a antibody (0.5 μg/mL; ab203698; Abcam, Cambridhe, UK) was used. The secondary antibody (Rhodamine Red-X donkey anti-rabbit IgG 711-295-152; Jackson Immunoresearch, Cambridgeshire, UK) was used at a 1:200 dilution. The nuclei were counterstained with DAPI, and the sections were mounted with a suitable mounting medium for fluorescence [62]. For analyses, slide images were acquired by a NanoZoomer S-60 digital slide scanner (NanoZoomer S60, Hamamatsu, Hamamatsu City, Japan). Several 10X fields were analyzed for each sample and morphological changes were graded semi-quantitatively according to the scale defined by Ashcroft [63] and modified by Hübner et al. [64] by two independent researchers blinded to the experimental design. An average Ashcroft ($\bar{x}$) was derived for each histological sample as a descriptive indication of the overall frames. In addition, the frequency percentage for each Ashcroft score was also reported, as presented in Table 1, so that the most frequent grade could be immediately identified for each sample analyzed.

### 3.6. Samples

After animal sacrifice, lung tissue samples were collected and histologically processed, and 19 formalin-fixed and paraffin-embedded (FFPE) tissue blocks were prepared. From each FFPE block, at least 5 tissue punches with a size of 2 × 3 mm were collected and stored at room temperature until the day of the analysis. At least 3 biological replicates (Table 1) for each group (saline, BLM 14 days, BLM 21 days, and NINT 21 days) were used for nLC-ESI-MS/MS analysis. In order to stratify the mice for the following proteomic analysis, both the average Ashcroft value ($\bar{x}$) and the micro-CT % poorly aerated tissue (*p*) were considered. Subjects were divided into 3 categories defined as mild ($\bar{x}$ ≤ 3.5 and *p* ≤ 25%), moderate ($\bar{x}$ > 3.5 or ≤4.5 and *p* > 25%), and severe ($\bar{x}$ > 4.5 and *p* > 50%) (Table 1).

### 3.7. nLC-ESI-MS/MS Sample Preparation and Analysis

Proteins were extracted from lung tissue punches for their identification and quantification by nLC-ESI-MS/MS [65]. Deparaffinization of the FFPE samples was performed by incubating the tissues for approximately 1 h at 65 °C, followed by 3 consecutive washes in toluene (4 min each while sonicating) and centrifugation (14,000× *g* rpm, 5 min). To ensure complete paraffin removal, these washing steps were repeated in triplicate. Subsequently, consecutive washes were performed while sonicating, in 100% (×2), 90%, and 70% of ethanol for 4 minutes each, as well as 100% water for 2 min, followed by centrifugation (14,000× *g* rpm, 5 min). Heat-induced antigen retrieval using a citrate buffer (10 mM, pH 6) at 97 °C for 45 min was performed, followed by centrifugation (14,000× *g* rpm, 5 min). Samples were washed while sonicating with 100% water for 2 min, followed by centrifugation (14,000× *g* rpm, 5 min). A 50 mM ammonium bicarbonate buffer solution was added to the samples, and, in order to enhance protein solubilization and digestion, RapiGestTM SF surfactant was added to each sample to a final concentration of 0.1%. After disulfide bond reduction (DTT 10 mM) and alkylation (IAA 15 mM), proteins were digested by adding 5 μg of trypsin (0.2 μg/μL), and samples were incubated overnight at 37 °C. The enzymatic reaction was stopped by acidification with TFA (pH < 2). Each sample was dried with a vacuum centrifugal evaporator (Hetovac, Savant) and resuspended in 50 µL of loading pump phase A (H_2_O:ACN:TFA 98:2:0.1), and protein content was determined by the NanoDrop assay (Thermo Scientific, Sunnyvale, CA, USA). Finally, desalting and concentration of the samples were performed using Ziptip™ µ-C18 pipette tips (Merck Millipore Ltd., Darmstadt, Germany), and, following the standard protocol provided by Millipore, peptides were eluted with a solution of 80% ACN and 0.1% FA, dried under vacuum using an Hetovac centrifuge, and resuspended in 50 µL of loading pump phase A. Tryptic peptides were then injected (volume of injection: 10 μL) into a Dionex UltiMate 3000 rapid separation (RS) LC nano system (Thermo Scientific, Germany), coupled on-line to an Impact HD™ Ultra High Resolution-QqTOF (Bruker Daltonics, Bremen, Germany). Each sample was injected in duplicate to minimize technical variability. Samples were loaded into a pre-column (Thermo Scientific, Acclaim PepMap 100, 100 µm × 2 cm, nanoViper, C18, 3 µm), followed by a 50 cm nano-column (Thermo Scientific, Acclaim PepMap RSLC, 75 µm × 50 cm, nanoViper, C18, 2 µm). The HPLC separation was performed at 40 °C and at a flow rate of 300 nL/min using a multistep gradient of 4 h from 4% to 98% of nanopump phase B (nanopump phase A being H_2_O w/0.1% FA and nanopump phase B being 80:20 ACN:H2O w/ 0.08% FA). The column was on-line interfaced to a nanoBoosterCaptiveSpray™ ESI source (Bruker Daltonics), where eluted peptides were ionized using heated nitrogen dry gas (T = 150 °C; 3 L/min) enriched with ACN. MS/MS spectra were generated by collision-induced dissociation, assisted by N2 functioning as a collision gas. Mass accuracy was improved by calibrating the instrument with a mix of ten standards with known masses (MMI-L Low Concentration Tuning Mix, Agilent Technologies, Santa Clara, CA, USA) by using a specific lock mass (*m*/*z* 1221.9906), and by an internal calibration based on a 15 min segment (10 mM sodium formate cluster solution) before the beginning of the gradient for every single run. Mass spectrometry data were acquired in data-dependent acquisition (DDA) modality, with automatic switching between full-scan MS and MS/MS acquisition. Acquisition parameters were set as already described [66].

### 3.8. nLC-ESI-MS/MS Data Elaboration

The Compass DataAnalysis v4.1 software (Bruker Daltonics, Hamburg, Germany) was used to calibrate, deconvolute, and convert the acquired raw data prior to protein identification and quantification. Peaks Studio X-Plus (Bioinformatics Solutions Inc., Waterloo, ON, USA) was used for protein identification and label-free quantification analysis. The parameters were set as follows: trypsin as the enzyme, carbamidomethyl as a fixed modification, oxidation (M), and FFPE+12 and FFPE+30 as variable modifications. The precursor mass error and the fragment mass error tolerances were set at 20 ppm and 0.05 Da, respectively. The identification engine uses an in-house-constructed UniProt reference database (accessed July 2021, 565,254 sequences; 203,850,821 residues), and the taxonomy of *Mus musculus* was selected (17,089 sequences). For identification, an FDR ≤ 1% was applied to all the analyses. Proteins were considered identified if there was at least one unique significant peptide (*p*-value ≤ 0.05). Label-free quantification analysis on Peaks Studio X-Plus was performed, and both quality and average abundance filters were applied and equal to 5 and 1 × 10^−5^, respectively. Proteins were selected for quantification if they were identified with at least 2 unique peptides. The list of quantified proteins was then imported into Microsoft Excel, and the statistical analysis was performed using in-house Excel add-in software. The area of the top 3-peptides was used to calculate the relative abundance of each protein. In order to investigate fibrosis progression and the effect of nintedanib treatment, the comparisons of moderate/mild, severe/mild, and moderate NINT/moderate BLM were evaluated. The quantified proteins were filtered using the non-parametric Mann–Whitney U-test corrected for multiple testing using a Benjamini–Hochberg adjusted *p*-value ≤ 0.05 and a fold-change (FC) ≥ 2 or ≤−2 in order to further investigate only those statistically significant and altered in fibrosis progression and drug-mediated slowdown. In order to further explore those pathways involved in fibrosis progression and drug mediated slow-down, the fold change was lowered to FC ≥ 1.5 or FC ≤ −1.5. Statistical analysis and exploratory data analysis were further investigated using Metaboanalyst 5.0 [67] and clustvis web tools [68]. Functional analysis of the comparisons was performed using the Cytoscape 3.9.1 network analysis platform. Gene ontology (GO) annotation, including biological process (BP) and molecular function (MF), as well as pathway term clustering using the Kyoto Encyclopedia of Genes and Genomes (KEGG) [69], were carried out using ClueGO 2.5.9 and CluePedia 1.5.9 [70]. Identifiers were UniProt accession numbers, and the organism reference database was *Mus musculus* (ClueGO mapped date 13 May 2022). For all of the analysis, the default parameters were used. The String-db open-source platform (https://string-db.org/ accessed on 17 September 2023) was used in order to investigate protein-protein interaction (PPI) networks.

## 4. Conclusions

This work represents a step forward in the comprehension and improved characterization of the bleomycin-induced pulmonary fibrosis mouse model. In particular, the proteomic fingerprint of fibrosis progression and response to nintedanib was unveiled from lung tissues. Although in this study a bulk analysis of lung tissue was performed, and spatial protein information was lost, our findings provide novel insights into the proteomic alterations involved in fibrosis progression and in drug-mediated slow-down. Coro1a could be considered as a putative biomarker of pulmonary fibrosis onset and progression. However, in the near future, further in vivo and in vitro studies are needed in order to determine Coro1a function and to decipher the underlying molecular mechanisms in lung fibrosis. Moreover, Ldhb expression, but not Ldha, was significantly restored during nintedanib treatment, suggesting Ldhb is a putative target of nintedanib action, but its role needs to be further investigated and validated in future research. The next step will be to integrate our nLC-ESI-MS/MS proteomics analysis with information regarding the spatial localization of proteins using single-cell matrix-assisted laser desorption/ionization mass spectrometry imaging (MALDI-MSI), allowing us to use more deeply investigate specific regions of interest (ROI) and hence to (i) explore the cell-to-cell communication jigsaw occurring during the fibrotic process; (ii) characterize the overall tissue proteomic profile based on the single microscope field histological fibrotic grade; and (iii) investigate the proteomic alterations related with different time courses after bleomycin treatment (i.e., comparing ROIs, having the same Ashcroft score, from samples exposed to BLM for 14 days with 21 days samples).

## Figures and Tables

**Table 1:** List of analyzed mice with their ID and treatment, average Ashcroft, Ashcroft frequencies, and fibrosis severity. Fibrosis severity was evaluated based on the average Ashcroft score: mild ( ≤ 3.5), moderate ( > 3.5 or ≤4.5), severe (> 4.5). * FFPE lung tissue blocks of these samples were prepared from both left and right lungs. SAL—saline; NINT—nintedanib; and BLM—bleomycin.

Animal ID and Treatment	Average Ashcroft	Ashcroft Frequencies									Fibrosis Severity
		0	1	2	3	4	5	6	7	8	
1856 (SAL)	1.19	0	80.6	19.4	0	0	0	0	0	0	Mild
1857 (SAL)	1.24	0	78	20	2	0	0	0	0	0	Mild
1860 (BLM 14d)	5.03	0	0	2.8	8.6	20	34.3	20	14.3	0	Severe
* 1862 (BLM 14d)	4.95	0	0	0	4.9	31.7	31.7	26.8	4.9	0	Severe
* 1864 (BLM 14d)	5.94	0	0	0	0	12.1	18.2	33.3	36.4	0	Severe
1869 (BLM 21d)	4.33	0	0	0	16.3	44.9	28.6	10.2	0	0	Moderate
1871 (BLM 21d)	5.4	0	0	0	2.1	27.7	19.1	29.8	21.3	0	Severe
1873 (BLM 21d)	3.94	0	0	0	34.3	48.6	5.7	11.4	0	0	Moderate
1874 (BLM 21d)	3.98	0	0	0	34.6	42.3	15.4	5.8	1.9	0	Moderate
1875 (BLM 21d)	3.92	0	0	0	41.7	36.1	11.1	11.1	0	0	Moderate
1891 (SAL)	1.68	0	41.2	50	8.8	0	0	0	0	0	Mild
1892 (SAL)	1.25	6.2	62.5	31.3	0	0	0	0	0	0	Mild
1893 (SAL)	0.85	21.7	71.7	6.6	0	0	0	0	0	0	Mild
1938 (NINT 21d)	3.77	0	0	0	40.9	43.2	13.6	2.3	0	0	Moderate
* 1941 (NINT 21d)	3.61	0	0	0	44.7	50	5.3	0	0	0	Moderate
* 1943 (NINT 21d)	4.66	0	0	3.1	12.5	21.9	46.9	9.4	6.2	0	Severe

## Data Availability

Data that support the findings of this study are available upon reasonable request from the corresponding author I.P.

## Supplementary Materials

The following supporting information can be downloaded at: https://www.mdpi.com/article/10.3390/ijms24054410/s1.
