# Supplementary material for: Proteomic Fingerprint of Lung Fibrosis Progression and Response to Therapy in Bleomycin-Induced Mouse Model
Source: Int J Mol Sci. 2023 Feb 23;24(5):4410. doi: 10.3390/ijms24054410 (PMC10002924; doi:10.3390/ijms24054410)
Supplement: Supplementary file 1 [file ijms-24-04410-s001.zip › ijms-2107202-supplementary.pdf]

Proteomic Fingerprint of Lung Fibrosis Progression and Response to Therapy in Bleomycin-Induced Mouse Model

**Lucrezia Principi [1,†], Erica Ferrini [2,†], Roberta Ciccimarra [2], Lisa Pagani [1], Clizia Chinello [1], Paolo Previtali [1],
Andrew Smith [1], Gino Villetti [3], Matteo Zoboli [2], Francesca Ravanetti [2], Franco Fabio Stellari [3,*], Fulvio Magni [1], and Isabella Piga [1,*]**

[1] Department of Medicine and Surgery, Clinical Proteomics and Metabolomics Unit, University of Milano-Bicocca, 20854 Monza, Italy; l.principi1@campus.unimib.it (L.P.); lisa.pagani@unimib.it (L.P.); clizia.chinello@unimib.it (C.C.); p.previtali1@campus.unimib.it (P.P.); andrew.smith@unimib.it (A.S.); fulvio.magni@unimib.it (F.M.)

[2] Department of Veterinary Science, University of Parma, 43122 Parma, Italy; erica.ferrini@unipr.it (E.F.); roberta.ciccimarra@unipr.it (R.C.); matteo.zoboli@unipr.it (M.Z.); francesca.ravanetti@unipr.it (F.R.)

[3] Experimental Pharmacology & Translational Science Department, Chiesi Farmaceutici S.p.A., 43122 Parma, Italy; g.villetti@chiesi.com (G.V.)

* Correspondence: fb.stellari@chiesi.com (F.F.S.); isabella.piga@unimib.it (I.P.)

† These authors contributed equally to this work.

Table S1: Assessment of lung function by micro-CT lung imaging.
List of analyzed mice with their ID and treatment, %Normo-aerated tissue and %Poorly aerated tissue.

Animal ID and Treatment	%Normo-aerated tissue	%Poorly-aerated tissue
1856 (SAL 14d)	81	19
1857 (SAL 14d)	85	15
1891 (SAL 21d)	82	18
1892 (SAL 21d)	82	18
1893 (SAL 21d)	87	13
1860 (BLM 14d)	39	61
1862 (BLM 14d)	36	64
1864 (BLM 14d)	33	67
1869 (BLM 21d)	73	27
1871 (BLM 21d)	30	70
1873 (BLM 21d)	74	26
1874 (BLM 21d)	45	55
1875 (BLM 21d)	71	29
1938 (NINT 21d)	53	47
1941 (NINT 21d)	61	39
1943 (NINT 21d)	48	52

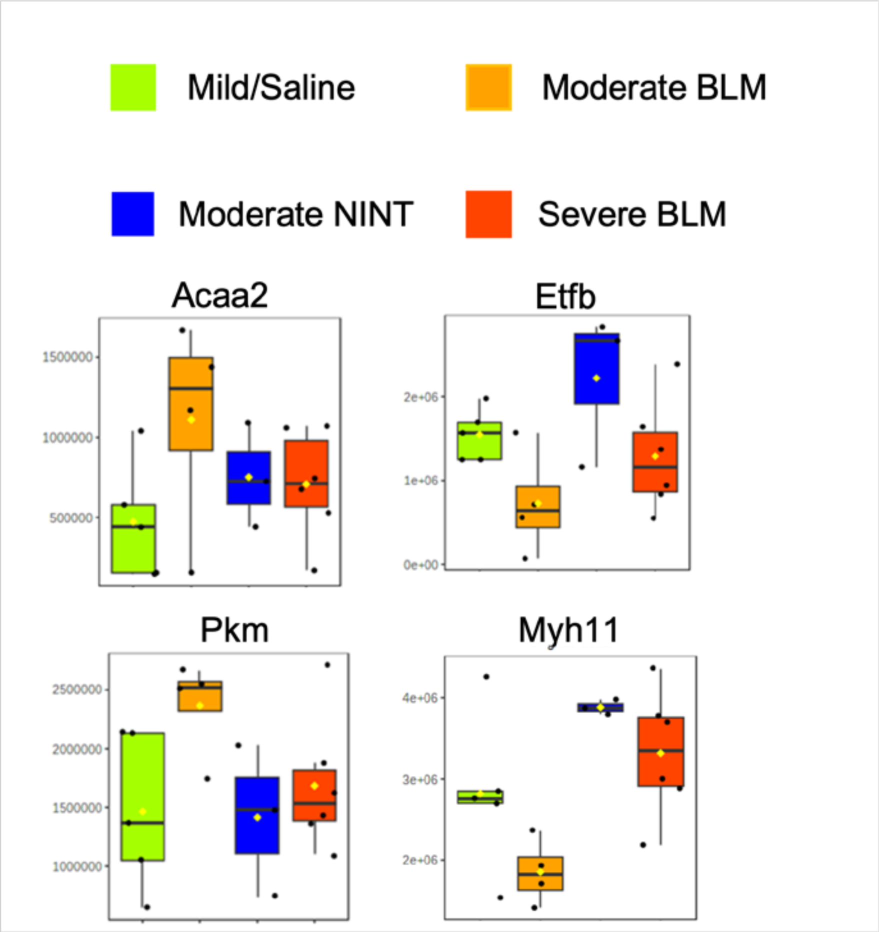

Figure S1: Box plots of 3-ketoacyl-CoA thiolase (Acaa2), electron transfer flavoprotein subunit beta (Etfb), pyruvate kinase (Pkm) and myosin heavy chain 11 (Myh11) showing their expression in mild (green), moderate BLM 21d (orange), moderate NINT 21d (blue) and severe BLM (dark orange). BLM: Bleomycin, NINT: Nintedanib, d: days.

Table S2: ANOVA Test of BLM samples mild, moderate and severe. Table obtained from statistical analysis on Metaboanalyst v.5.0.

	**f.value**	**p.value**	**LOG10(p)**	**FDR**	**Fisher's LSD**					
Ldhb	39.073	5.56E-06	5.2546	0.001334	mild - Moderate BLM					mild - severe BLM
Coro1a	35.01	9.81E-06	5.0084	0.001334		Moderate BLM - mild	severe BLM - Moderate BLM	severe BLM - mild		
Rpl7a	28.375	2.83E-05	4.5486	0.002287			severe BLM - Moderate BLM	severe BLM - mild		
Fmo1	27.397	3.36E-05	4.4733	0.002287	mild - Moderate BLM					mild - severe BLM
Anxa1	24.291	6.04E-05	4.219	0.002925		Moderate BLM - mild		severe BLM - mild		
Des	23.959	6.45E-05	4.1902	0.002925			severe BLM - Moderate BLM	severe BLM - mild		
Fn1	22.704	8.34E-05	4.0787	0.003242			severe BLM - Moderate BLM	severe BLM - mild		
Rpl29	21.672	0.00010392	3.9833	0.003533		Moderate BLM - mild	severe BLM - Moderate BLM	severe BLM - mild		
Rras2	18.615	0.00020976	3.6783	0.006339	mild - Moderate BLM					mild - severe BLM
Cd36	17.98	0.00024536	3.6102	0.006674	mild - Moderate BLM					mild - severe BLM
Fgg	17.145	0.0003035	3.5178	0.007484			severe BLM - Moderate BLM	severe BLM - mild		
Fga	16.611	0.0003491	3.4571	0.007484			severe BLM - Moderate BLM	severe BLM - mild		

Rpl7	16.52	0.00035767	3.4465	0.007484			severe BLM - Moderate BLM	severe BLM - mild		
Cndp2	15.424	0.00048255	3.3165	0.009375		Moderate BLM - mild		severe BLM - mild		
Hnrnpa2b1	14.098	0.00070788	3.15	0.01205			severe BLM - Moderate BLM	severe BLM - mild		
Eif4b	13.932	0.00074402	3.1284	0.01205			severe BLM - Moderate BLM	severe BLM - mild		
Nme2	13.787	0.0007774	3.1094	0.01205		Moderate BLM - mild		severe BLM - mild		
Fgb	13.644	0.00081191	3.0905	0.01205			severe BLM - Moderate BLM	severe BLM - mild		
Rpl18	13.526	0.00084175	3.0748	0.01205		Moderate BLM - mild		severe BLM - mild		
Cbr2	12.77	0.0010669	2.9719	0.013889	mild - Moderate BLM					mild - severe BLM
Rnh1	12.754	0.0010723	2.9697	0.013889		Moderate BLM - mild		severe BLM - mild		
Aldh1a1	11.575	0.0015829	2.8005	0.019571	mild - Moderate BLM					mild - severe BLM
Efemp1	11.343	0.0017146	2.7658	0.020277	mild - Moderate BLM					mild - severe BLM
Anxa2	10.976	0.0019496	2.7101	0.021625		Moderate BLM - mild		severe BLM - mild		
Trf	10.867	0.002026	2.6934	0.021625		Moderate BLM - mild			Moderate BLM - severe BLM	
Rps9	10.759	0.0021055	2.6766	0.021625			severe BLM - Moderate BLM	severe BLM - mild		

Lcp1	10.632	0.0022043	2.6567	0.021625		Moderate BLM - mild		severe BLM - mild		
Myh14	10.604	0.0022262	2.6524	0.021625					Moderate BLM - severe BLM	mild - severe BLM
Snx2	10.299	0.0024884	2.6041	0.022936		Moderate BLM - mild		severe BLM - mild		
Cct8	10.254	0.0025297	2.5969	0.022936		Moderate BLM - mild		severe BLM - mild		
Map4	10.119	0.0026602	2.5751	0.023341			severe BLM - Moderate BLM	severe BLM - mild		
Eif3b	9.8638	0.0029273	2.5335	0.024882			severe BLM - Moderate BLM	severe BLM - mild		
Rab10	9.5658	0.00328	2.4841	0.027035	mild - Moderate BLM					mild - severe BLM
Col4a1	8.9631	0.0041569	2.3812	0.033255	mild - Moderate BLM					mild - severe BLM
Anxa4	8.6408	0.0047371	2.3245	0.036814		Moderate BLM - mild		severe BLM - mild		
Arpc1b	8.5478	0.0049218	2.3079	0.037187		Moderate BLM - mild		severe BLM - mild		
Rpl13a	8.4263	0.0051758	2.286	0.038049			severe BLM - Moderate BLM	severe BLM - mild		
Rrbp1	8.344	0.0053566	2.2711	0.038342			severe BLM - Moderate BLM	severe BLM - mild		
Elavl1	8.2631	0.0055414	2.2564	0.038473			severe BLM - Moderate BLM	severe BLM - mild		
Ckmt1	8.2098	0.0056674	2.2466	0.038473		Moderate BLM - mild		severe BLM - mild		

Vim	8.1122	0.0059066	2.2287	0.038473			severe BLM - Moderate BLM	severe BLM - mild		
Ehd2	8.0987	0.0059407	2.2262	0.038473	mild - Moderate BLM					mild - severe BLM
Hnrnpm	7.7713	0.0068401	2.1649	0.043267			severe BLM - Moderate BLM	severe BLM - mild		
Lamb3	7.6708	0.0071473	2.1459	0.044183	mild - Moderate BLM					mild - severe BLM
Psmb10	7.5842	0.0074251	2.1293	0.044876			severe BLM - Moderate BLM	severe BLM - mild		
Lyz2	7.4894	0.0077437	2.1111	0.044876					Moderate BLM - severe BLM	
Aldh6a1	7.4052	0.0080404	2.0947	0.044876			severe BLM - Moderate BLM	severe BLM - mild		
Rps3a	7.4027	0.0080493	2.0942	0.044876		Moderate BLM - mild		severe BLM - mild		
Cdv3	7.3787	0.0081362	2.0896	0.044876				severe BLM - mild		
Ager	7.3318	0.0083096	2.0804	0.044876						mild - severe BLM
Rbm3	7.304	0.0084143	2.075	0.044876			severe BLM - Moderate BLM	severe BLM - mild		
Selenbp1	7.1424	0.0090542	2.0431	0.04736	mild - Moderate BLM					mild - severe BLM
Mdh2	7.0066	0.0096365	2.0161	0.049455	mild - Moderate BLM					mild - severe BLM

Table S3: Proteins with a statistically significant correlation in BLM samples mild, moderate and severe fibrosis. The correlation values are expressed respect to the pattern search 1-2-3 (proteins with an increasing trend from mild to severe fibrosis). Table obtained from statistical analysis on Metaboanalyst v.5.0.

	correlation	t-stat	p-value	FDR
Coro1a	0.92326	8.665	9.23E-07	0.000251
Rpl7a	0.88627	6.8991	1.09E-05	0.001091
Rpl29	0.88439	6.8318	1.20E-05	0.001091
Fn1	0.86399	6.187	3.29E-05	0.001788
Rpl7	0.848	5.7688	6.50E-05	0.002527
Fgg	0.83978	5.5769	8.97E-05	0.002662
Eif4b	0.83286	5.4254	0.000116	0.002662
Hnrnpa2b1	0.83187	5.4045	0.00012	0.002662
Rpl18	0.83005	5.3665	0.000128	0.002662
Des	0.82982	5.3617	0.000129	0.002662
Nme2	0.82851	5.3346	0.000136	0.002662
Fgb	0.82651	5.2938	0.000145	0.002662
Fga	0.82624	5.2883	0.000147	0.002662
Cndp2	0.82347	5.2332	0.000162	0.002746
Rnh1	0.78889	4.6286	0.000473	0.006177
Rps9	0.78713	4.6013	0.000497	0.006177
Eif3b	0.78682	4.5965	0.000501	0.006177
Map4	0.78531	4.5734	0.000522	0.006177
Anxa1	0.77208	4.3803	0.000744	0.007785
Anxa2	0.75579	4.1616	0.001117	0.011197
Elavl1	0.75299	4.1259	0.001194	0.011197
Hnrnpm	0.74451	4.0208	0.001454	0.012189
Rpl13a	0.74025	3.9698	0.001601	0.012189
Vim	0.74	3.9668	0.00161	0.012189
Cdv3	0.7399	3.9657	0.001613	0.012189
Rbm3	0.73332	3.8889	0.001865	0.013709
Psmb10	0.72839	3.8331	0.002073	0.014433
Anxa4	0.72226	3.7653	0.002358	0.015643
Myl6	0.72042	3.7453	0.002449	0.015818
Rrbp1	0.71941	3.7344	0.002501	0.015818
Ckmt1	0.7066	3.6003	0.00323	0.018324
Rpl4	0.70511	3.5853	0.003324	0.018451
Rps3a	0.69976	3.5318	0.003683	0.020033
Sfpq	0.69017	3.4387	0.004402	0.023479
Arpc1b	0.68252	3.367	0.005053	0.026428
Rpl6	0.68023	3.346	0.005261	0.026821
Cct8	0.67722	3.3186	0.005546	0.027426
Pdia3	0.67356	3.2857	0.005908	0.02749
Rps25	0.6735	3.2851	0.005915	0.02749

Palld	0.67319	3.2824	0.005946	0.02749
Fbn1	0.66582	3.2175	0.006736	0.030538
Psmc2	0.65663	3.1391	0.007835	0.033826
Uap1l1	0.65546	3.1292	0.007984	0.033933
Lcp1	0.64808	3.0682	0.00898	0.037576
Eef2	0.64439	3.0383	0.009511	0.039197
Tardbp	0.63369	2.9535	0.011195	0.044839
Rps18	0.63361	2.9529	0.01121	0.044839
Eef1a1	0.62595	2.894	0.012552	0.048201
Sarnp	0.62525	2.8887	0.012681	0.048201
Rpl8	0.62465	2.8841	0.012792	0.048201
Pgls	0.62388	2.8783	0.012936	0.048201
Rps3	0.62225	2.866	0.013245	0.048684
Col6a2	0.61724	2.8286	0.014228	0.049802
Aldoa	0.61445	2.808	0.014801	0.049802
Snx2	0.61388	2.8039	0.014918	0.049802
Rps19	0.61221	2.7916	0.015272	0.049802
Anxa7	0.61167	2.7877	0.015387	0.049802
Lmna	0.61013	2.7765	0.015721	0.049802
Plec	0.61001	2.7757	0.015746	0.049802
Pa2g4	0.60838	2.7639	0.016105	0.05035
Tnks1bp1	0.6025	2.7219	0.017451	0.05394
Pdlim5	0.60084	2.7101	0.017848	0.054545
Rps6	0.5997	2.702	0.018124	0.054775
Bgn	0.59881	2.6958	0.018341	0.054821
Flna	0.59655	2.68	0.018902	0.055884
Lmnb1	0.59084	2.6404	0.020379	0.058968
Pfn1	0.58864	2.6254	0.02097	0.06004
P4hb	0.58311	2.5879	0.022516	0.063795
Aldh6a1	0.57572	2.5387	0.024714	0.0693
Eef1d	0.57246	2.5173	0.025733	0.071422
Ldha	0.56754	2.4853	0.027334	0.075098
Rpl11	0.5589	2.4301	0.030323	0.081662
Vat1	0.54894	2.3679	0.034069	0.088255
Lasp1	0.54331	2.3334	0.036335	0.092556
Pabpc1	0.53544	2.2858	0.039687	0.098177
Efemp2	0.52564	2.2278	0.044178	0.10729
Ehd4	-0.53104	-2.2597	0.041657	0.10208
Anxa5	-0.5354	-2.2856	0.039704	0.098177
Cyp2f2	-0.53884	-2.3063	0.038211	0.096235
Limch1	-0.54313	-2.3323	0.03641	0.092556
Hspg2	-0.54906	-2.3686	0.034024	0.088255
Ywhab	-0.55365	-2.3972	0.032256	0.08518
Scgb1a1	-0.55468	-2.4035	0.031873	0.084994
Myo1c	-0.5593	-2.4326	0.030182	0.081662
H1-1	-0.59181	-2.6472	0.02012	0.058845

Mdh2	-0.61143	-2.786	0.015439	0.049802
Lamc2	-0.61379	-2.8032	0.014938	0.049802
Ctnnd1	-0.61452	-2.8086	0.014785	0.049802
Crip2	-0.61589	-2.8186	0.014503	0.049802
Lama3	-0.61726	-2.8288	0.014224	0.049802
Spr	-0.62708	-2.9025	0.012347	0.048201
Dpysl2	-0.66216	-3.186	0.007158	0.031401
Lamb2	-0.6639	-3.2009	0.006956	0.031015
Itgb1	-0.67302	-3.2809	0.005963	0.02749
Aldh2	-0.67955	-3.3397	0.005325	0.026821
Prdx6	-0.70653	-3.5997	0.003234	0.018324
Rap1a	-0.71536	-3.6912	0.002715	0.016056
Rap1b	-0.71536	-3.6912	0.002715	0.016056
Selenbp1	-0.71728	-3.7116	0.002612	0.016056
Rab10	-0.72728	-3.8207	0.002122	0.014433
Lamb3	-0.73055	-3.8573	0.00198	0.014171
Ager	-0.7415	-3.9847	0.001556	0.012189
Col4a1	-0.74192	-3.9896	0.001542	0.012189
Cbr2	-0.74237	-3.995	0.001526	0.012189
Ehd2	-0.75315	-4.1278	0.00119	0.011197
Rras2	-0.77336	-4.3983	0.00072	0.007785
Aldh1a1	-0.78229	-4.5281	0.000567	0.00643
Myh14	-0.78732	-4.6043	0.000494	0.006177
Ldhb	-0.80418	-4.8781	0.000302	0.004558
Efemp1	-0.80738	-4.9338	0.000273	0.004371
Cd36	-0.85536	-5.9533	4.8E-05	0.002175
Fmo1	-0.86557	-6.2318	3.06E-05	0.001788

Table S4: Proteins with a statistically significant correlation in BLM samples mild, moderate and severe fibrosis. The correlation values are expressed respect to the pattern search 1-2-1 (proteins high only in moderte fibrosis). Table obtained from statistical analysis on Metaboanalyst v.5.0.

	correlation	t-stat	p-value	FDR
Ptms	0.51836	21,855	0.04775	0.90856
Tkt	-0.53628	-22,909	0.039319	0.90856
Pkm	0.55732	24,202	0.030895	0.90856
Ddah2	-0.58119	-2,575	0.023072	0.89653
Myh11	-0.60264	-27,228	0.017419	0.78965
Hspb1	-0.61768	-28,319	0.014139	0.76919
Lyz2	0.61832	28,367	0.01401	0.76919
Alb	0.63874	29,932	0.010374	0.76919
Atp6v1a	-0.68303	-33,718	0.0050066	0.6809
Trf	0.80248	48,491	0.00031766	0.086404

Table S5: Differentially expressed proteins in the pairwise comparisons severe/mild and moderate/mild of BLM mice samples (p-value$_{adj}$≤ 0.05 and with a fold-change (FC) ≥ 2 or ≤ -2). Comparison of our proteomic dataset based on BLM mouse model with the multi-omics studies on human IPF subjects of Zheng et al. [9] and Konigsberg et al. [10].

	BLM Mouse model				Human			
					Zheng et al. [9]		Konigsberg et al. [10]	
	Proteomics				Trascriptomics	Proteomics	polyA RNA-seq (coding RNA)	Proteomics
	Severe/mild		Moderate/mild		end stage IPF/healthy donors		IPF/controls	
Gene name	FC	LOG2FC	FC	LOG2FC	LOG2FC	LOG2FC	LOG2FC	LOG2FC
Fn1	4.96	2.31	2.28	1.19				
Cbr2	-4.23	-2.08	-3.35	-1.75				
Fgb	11.86	3.57	5.04	2.33				-1.27
Aldh1a1	-6.10	-2.61	-3.05	-1.61				-0.69
Fgg	7.40	2.89	3.28	1.71				-1.20
Fga	10.22	3.35	4.07	2.02				
Coro1a	3.21	1.68						
Rbm3	3.95	1.98	2.08	1.06				
Ckmt1	5.26	2.40	4.73	2.24				
Snx2	3.74	1.90	4.60	2.20				
Rps6	3.98	1.99	4.68	2.23			-2.04	
Uap1l1	2.46	1.30						
Rpl4	3.16	1.66	2.72	1.44			-0.70	
Eif3b	3.36	1.75	2.28	1.19				
Rps3a	2.69	1.43	3.04	1.60				
Rpl7	2.97	1.57						-1.46
Vim	2.29	1.20						0.65

Anxa1	3.10	1.63	3.12	1.63				
Eif4b	2.96	1.57	2.13	1.09				
Fmo1	-6.16	-2.62	-2.67	-1.42				1.61
Ldhb			-3.47	-1.80				
Rpl11			3.16	1.66				
Rpl29	2.45	1.29					-0.82	
Rrbp1	2.29	1.19					-1.35	
Ldhb	-3.31	-1.73						
Rpl11	3.15	1.66						
Srsf1	2.17	1.11	2.48	1.31			-1.90	
Anxa3			2.16	1.11				-1.35
Hspa8			2.42	1.27				0.55
Cdv3	2.91	1.54	2.55	1.35			-0.93	
Pgls	2.34	1.23	2.41	1.27			1.05	
Tnks1bp1	2.51	1.33					-1.01	
Rpl6	3.07	1.62	3.35	1.74			1.11	-1.02
Rras2	-2.98	-1.58	-2.67	-1.42				
Atp5po			2.18	1.12				
Nme2	2.04	1.03						-0.98
Pdia3	3.23	1.69	3.17	1.67				
Efemp1	-2.37	-1.24					-1.18	0.71
Prdx6	-2.14	-1.10						
Map4	2.00	1.00					-1.48	
Ager	-2.70	-1.44			-3.16	-4.55	-2.79	-4.18
Des	2.20	1.14						1.17
Rps3	2.29	1.20	2.26	1.18				0.86
Rap1a	-2.84	-1.50						
Rap1b	-2.84	-1.50						

Inmt	-3.25	-1.70						
Ehd2	-2.41	-1.27					-1.26	-0.95
Ldha	2.19	1.13						0.45
Cd36	-2.84	-1.50						-2.16
Ehd4	-2.48	-1.31					-0.90	-0.71
Rps18	2.02	1.01						0.93
Anxa4	3.49	1.80	2.99	1.58			1.50	
Vat1	2.65	1.40						
Selenbp1	-2.59	-1.37			-1.46	-1.25		-0.56
Eln	2.29	1.20						
Khsrp	3.48	1.80	2.50	1.32			-0.86	0.59
Myo1c	-2.11	-1.07					-1.36	
Cct8	2.01	1.01					-0.66	
Srsf3	2.45	1.29	4.97	2.31			-1.73	
Pdlim5	4.03	2.01	3.52	1.82			-1.96	
Tardbp	15.97	4.00	18.77	4.23			-0.71	
Hdlbp	2.85	1.51	2.97	1.57			-0.64	-0.97
Scgb1a1	-2.13	-1.09						
Rps16	3.70	1.89	5.07	2.34			-1.26	
Cyp2f2	-2.46	-1.30						
Krt4	-8.80	-3.14	-9.80	-3.29				
Rps9	2.51	1.33						
Psmc2	2.23	1.15						
Limch1	-2.73	-1.45			-1.05	-0.88	3.53	
Anxa7	2.62	1.39	2.71	1.44				
Dpt	2.67	1.42	2.77	1.47				
Palld	2.44	1.29						1.11
Efemp2	2.72	1.44						

Idh2	-2.71	-1.44	-2.70	-1.43			-0.73	
Lamc2	-2.56	-1.36	-2.20	-1.13				-1.61
Ctnnd1	-2.73	-1.45					-1.87	-0.95
Add1	-2.39	-1.26						
Rac1	2.05	1.03					-0.86	
Etfb	-2.46	-1.30					1.40	
Actg1			2.21	1.14			-1.29	
Sptbn1			2.10	1.07			-1.54	-0.62
Col4a2			2.19	1.13			-3.38	-1.16
Uba1			2.56	1.36			-0.90	
Eef1a1			2.16	1.11			-0.83	
Uqcrc2			-5.60	-2.48			1.66	0.53
Eln			3.09	1.63				
Sh3bgrl			2.30	1.20				
Lcp1			2.16	1.11				
Tf			2.06	1.04				
Prx			2.14	1.09			-1.77	
Rpn1			3.28	1.71				
Krt18			2.02	1.02			2.17	
Hsp90ab1			2.40	1.26			-1.68	
Ehd1			3.15	1.66				-0.81
Acaa2			2.98	1.58				
Ddah2			-2.09	-1.06			-3.07	
Aldh6a1			-2.01	-1.01				
